# Mouse Mesonephros Promotes Differentiation of Oogonial Stem Cells into Follicle-like Structures via the PI3K/AKT Signaling Pathway

**DOI:** 10.3390/ijms27198719

**Published:** 2026-09-29

**Authors:** Jie Chen, Ziyao Wang, Rui Xiao, Shaojie Zhang, Yunteng Hao, Xing Wang

**Affiliations:** 1 College of Basic Medicine, Inner Mongolia Medical University, Hohhot 010000, China; 2 Digital Medical Center, Inner Mongolia Medical University, Hohhot 010000, China

**Keywords:** mesonephros, OSCs, Foxl2, Lgr5, Gli1

## Abstract

While the mesonephros persists in the adult mouse ovary and contributes to folliculogenesis, its inductive role in oogonial stem cell (OSC) differentiation remains uncertain. In this study, we investigated whether mesonephros-derived cells direct OSCs toward follicle-like structures in vitro. OSCs were co-cultured with mesonephros cells, and morphological assessments and factor expression analyses were conducted at multiple time points. On day 14 of co-culture, 10× Genomics single-cell and bulk RNA sequencing were performed to profile transcriptomic changes, while renal capsule transplantation was conducted to evaluate the functional potential of co-cultured clusters in recipient mice. Transcriptomic analyses revealed a robust expression of mesonephros-specific genes and a significant enrichment of PI3K/AKT pathway-associated genes in co-cultured cells. Notably, transplanted clusters exhibited signs of estrogen secretion with maintained graft integrity. Collectively, these findings demonstrate that the mesonephros promotes OSC differentiation into follicle-like structures, with the PI3K/AKT pathway identified as a candidate mediator of this process. Our study provides novel insights into the developmental regulation of ovarian folliculogenesis and offers a valuable experimental model for future investigations.

## 1. Introduction

The discovery of stem cells that exhibit proliferative activity in mouse ovaries by Johnson and Wu Ji gave rise to the concept of ovarian stem cells (OSCs) [1,2]. In the ovary, a very small number of reproductive stem cells possess the potential for self-renewal and differentiation, enabling them to generate new oocytes. This characteristic is essential for replenishing the follicular pool, improving fertility, and paving new paths for the study of granulosa cells [3]. Interstitial dilatation mainly occurs during the initial stages of ovarian development [4]. When the mesonephric interstitium (containing fibroblasts, fibers, and capillaries) permeates the reproductive crest, these genes are highly expressed, giving rise to follicular cords containing epithelioid cells (GREL) and oogonia [5,6].

The development and differentiation of follicles depend on the support of ovarian cells, which provide necessary regulatory factors for follicle survival and development [7]. Granulosa cells and ovarian membrane cells are important components of ovarian cells [8]. As the largest cell group in ovarian cells, ovarian granulosa cells are the primary source of estrogen and progesterone secretion, playing a vital role in the development of oocytes and embryos [9,10,11]. Meanwhile, theca cells contain a variety of cell populations [12] that directly or indirectly affect the growth and development of follicles and are essential for follicle genesis, proliferation, and differentiation [13]. We have previously successfully completed the isolation, establishment, and in vitro differentiation of mouse OSCs and have conducted relevant experiments on the co-culturing of OSCs and ovarian cells to form functional oocytes [14].

The mesonephros, despite being a transient organ during fetal development [13], has increasingly been recognized as a critical source of ovarian somatic cells. In 1991, M. SATOH [8] investigated the gonadal development process of 5–18-week-old human embryos using the method of continuous semi-thin sections and concluded that some key cells in the human gonads originate from the mesonephros. Subsequent studies have shown that surface epithelial cells of the mesonephros undergo a series of developmental processes, ultimately transforming into germinal epithelial cells, which then differentiate into surface epithelial stem cells and pregranulosa cells [15]. During follicular maturation, granulosa cells differentiate into two main groups [16]: one group consists of granulosa cells that envelop oocytes and are labeled with forkhead box L2(Foxl2) in the embryonic ovary, while the other group includes granulosa cells that are co-labeled with Foxl2 and Leucine-rich G-protein-coupled receptor 5 (Lgr5)-positive cells from the ovarian epithelium in the follicular wall layer [17,18]. Follicular cells are made up of germ cells and somatic cells [19], with somatic cells including granulosa cells (cumulus granulosa cells and parietal granulosa cells) and theca cells located at the outermost layer of the follicle [20,21]. Notably, theca cells originate from membrane progenitor cells [22], with Gli1-positive mesenchymal cells migrating from the mesonephros serving as an important source of ovarian theca progenitor cells [23]. These Gli1-positive cells first appear in the medulla of neonatal ovaries and play a crucial role in the development of the first wave of follicles.

The phosphatidylinositol 3-kinase (PI3K)/protein kinase B (AKT) signaling pathway has been reported to directly or indirectly promote follicle genesis and development [24]. As a protein kinase, AKT is an important signaling molecule downstream of PI3K, widely distributed in normal tissues. It is critical for cell survival and promotes cell proliferation, invasion, and migration [25]. Vascular endothelial growth factor (VEGF), which mainly functions as a ligand activating PI3K/AKT through VEGFR2, plays a key role in establishing blood supply in follicular microcirculation and improving nutrient supply [26].

The reasons for choosing to co-culture OSCs and mesonephros are as follows: first, the mesonephros is a known source of ovarian somatic progenitors [8,15,23]; second, Gli1-positive cells participate in the first wave of follicle development; finally, the direct inductive capacity of the mesonephros on OSCs remains untested. We therefore hypothesized that mesonephros-derived cells would promote OSC differentiation in vitro, and our subsequent transplantation experiment was designed to assess whether the in vitro-formed structures possess functional competence.

Despite these advances, several critical questions remain unanswered: the direct inductive role of the mesonephros in promoting OSC differentiation toward follicle-like structures has not been experimentally tested, and the molecular mechanisms underlying such mesonephros-mediated effects remain largely unexplored. In the present study, we address these gaps by examining whether mesonephros-derived cells direct OSCs toward follicle-like structures in vitro and identifying the potential signaling pathways involved. The novelty of our work lies in (1) establishing a co-culture model that recapitulates mesonephros–OSC interaction; (2) providing transcriptomic evidence identifying the PI3K/AKT pathway as a candidate mediator; and (3) demonstrating the functional potential of differentiated OSC clusters via in vivo transplantation. Our findings offer new perspectives on folliculogenesis and provide a valuable experimental platform for future ovarian biology studies.

## 2. Results

### 2.1. Morphological Observation of Follicle-like Structures Through HE Staining

**Figure 1:**
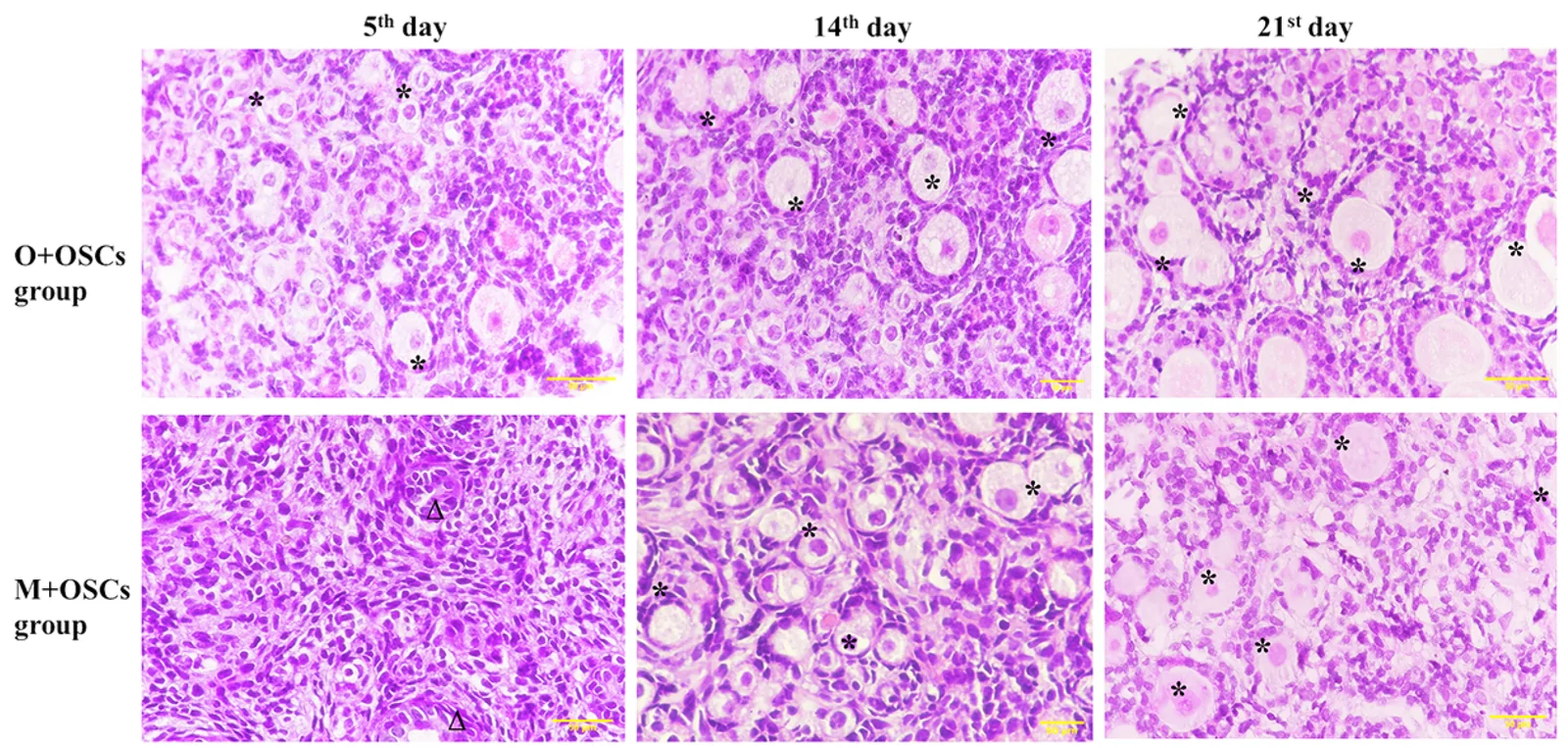
Morphological observation of HE staining in co-cultured cell clusters (50 μm). * represents follicle-like structures and Δ represents mesonephric ducts. Data are presented as mean ± SD (*n* = 20 per group). Statistical significance was determined by one-way ANOVA followed by LSD post hoc test, *p* < 0.05.

To dynamically observe the morphological development of follicles during co-culture, hematoxylin and eosin (HE) staining was conducted at three key time points: days 5, 14, and 21 after co-culture initiation. The histological results revealed distinct temporal and group-specific differences in follicle-like structure formation and development between the O + OSC and M + OSC groups. Specifically, at the early co-culture stage (day 5), preliminary follicle-like structures were exclusively detected in the O + OSC group, while the M + OSC group only presented regular and intact tissue structural characteristics without the formation of distinct follicular structures, indicating an earlier follicle-like structure initiation in the O + OSC group. On day 14 of co-culture, follicle-like structure formation was further promoted in the O + OSC group, with a prominent increase in the number of follicle-like structures. Notably, a large number of follicle-like structures, primarily at the primordial follicle stage, emerged in the M + OSC group at this time point, showing robust follicle-like structure developmental potential. However, obvious group differences in follicle-like structure growth dynamics were observed at the late culture stage (day 21); while the quantity of follicle-like structures in the O + OSC group continued to rise steadily, the M + OSC group exhibited a significant reduction in the number of follicle-like structures relative to its day 14 level, and this difference was statistically significant (*p* < 0.05) (Figure 1) (Table 1).

### 2.2. Dynamic Observation of Follicle-like Structure Formation and Mesonephros Cell Migration Using Live-Cell Imaging

**Figure 2:**
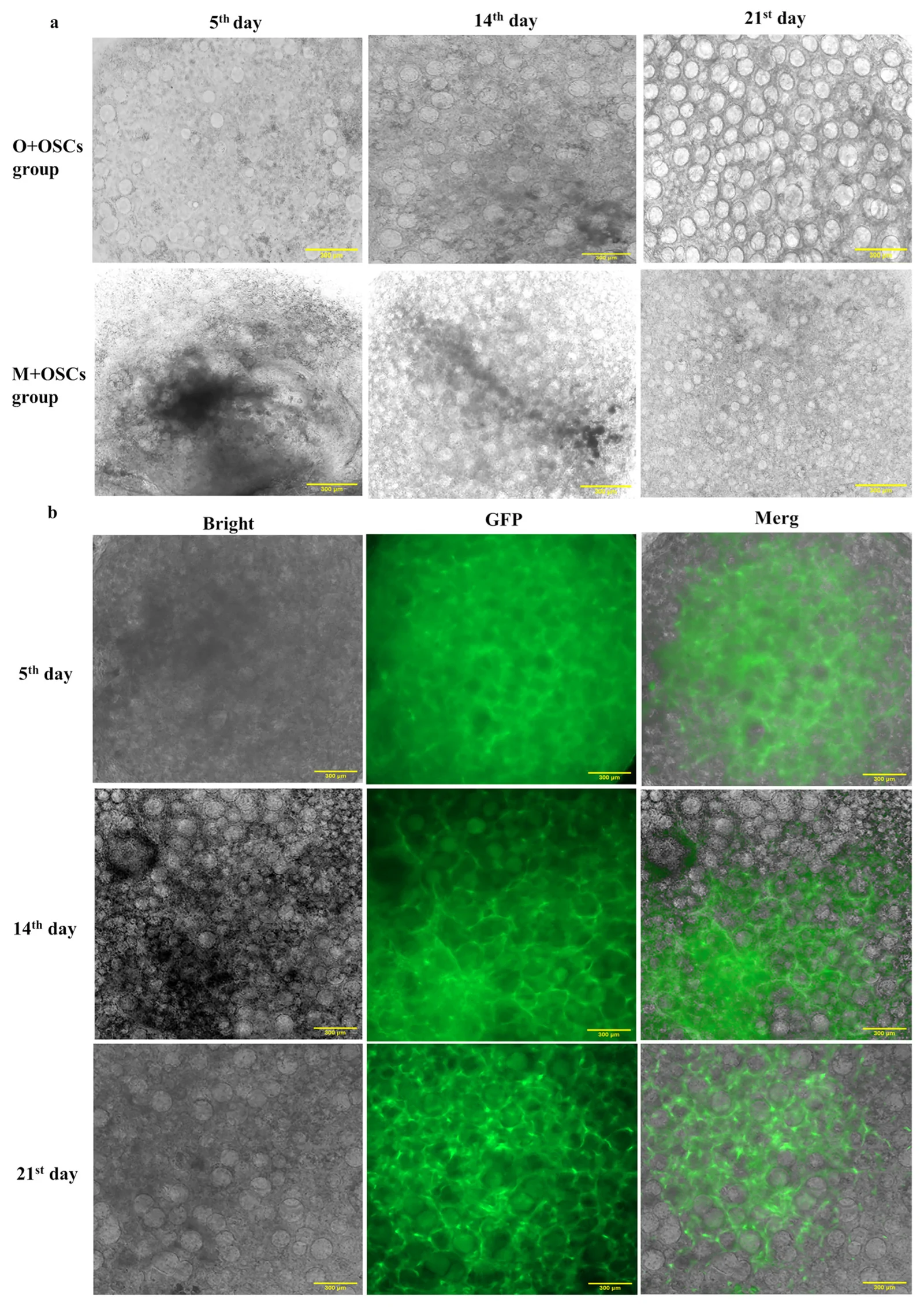
Live-cell workstation tracing of mesonephros cell migration (600×, scale bar: 300 μm). (**a**) Morphological changes in the two co-culture groups (O + OSC and M + OSC) on days 5, 14, and 21. (**b**) Migration of GFP-labeled mesonephros cells in the M + OSC group at days 5, 14, and 21. All images were captured at identical magnification; the apparent size differences in cell clusters across time points reflect their progressive growth and developmental dynamics during in vitro culture. GFP-positive cells indicate mesonephros-derived cells. Data are presented as mean ± SD (*n* = 20 per group). Statistical significance was determined by one-way ANOVA followed by LSD post hoc test, *p* < 0.05.

To further characterize the dynamic developmental characteristics of follicle-like structures in different co-culture systems and validate the morphological differences observed via HE staining, dynamic morphological changes in follicle-like structures in the O + OSC and M + OSC groups were continuously monitored via live-cell workstation imaging at days 5, 14, and 21 of co-culture. Consistent with the histological staining results, continuous live-cell observation showed that abundant follicle-like structures were stably present in the O + OSC group at both day 14 and day 21 of co-culture. By comparison, follicle-like structure formation in the M + OSC group exhibited a delayed initiation pattern, with distinct follicle-like structures first emerging at day 14. Although follicle-like structures persisted in the M + OSC group at the late culture stage (day 21), these follicle-like structures displayed markedly smaller morphological sizes relative to those in the O + OSC group at the same time point (Figure 2a). To further trace the dynamic behavior and contribution of mesonephros cells during follicle development in the M + OSC co-culture system, we performed cell lineage tracing using GFP-labeled mesonephros cells. Serial live-cell imaging across the three detection time points demonstrated that GFP-positive mesonephros cells underwent active migration throughout the formation of follicle-like structures. The consistent detection of GFP-expressing follicle-like structures at different co-culture stages provided direct visual evidence confirming the regulatory role of mesonephros cells in facilitating in vitro follicular differentiation (Figure 2b). Quantitative statistical analysis further verified the significant temporal differences in the number of GFP-positive follicle-like structures in the M + OSC group. Specifically, the abundance of GFP-positive follicle-like structures peaked at day 14, which was significantly higher than that at day 5 and day 21 (*p* < 0.05). Despite a noticeable decline in the number of GFP-positive follicle-like structures at day 21, their quantity remained substantially higher than the baseline level observed at day 5 (*p* < 0.05) (Table 2).

### 2.3. Expression of Granulosa Cell Markers (Foxl2 and Lgr5) and Theca Progenitor Marker (Gli1)

**Figure 3:**
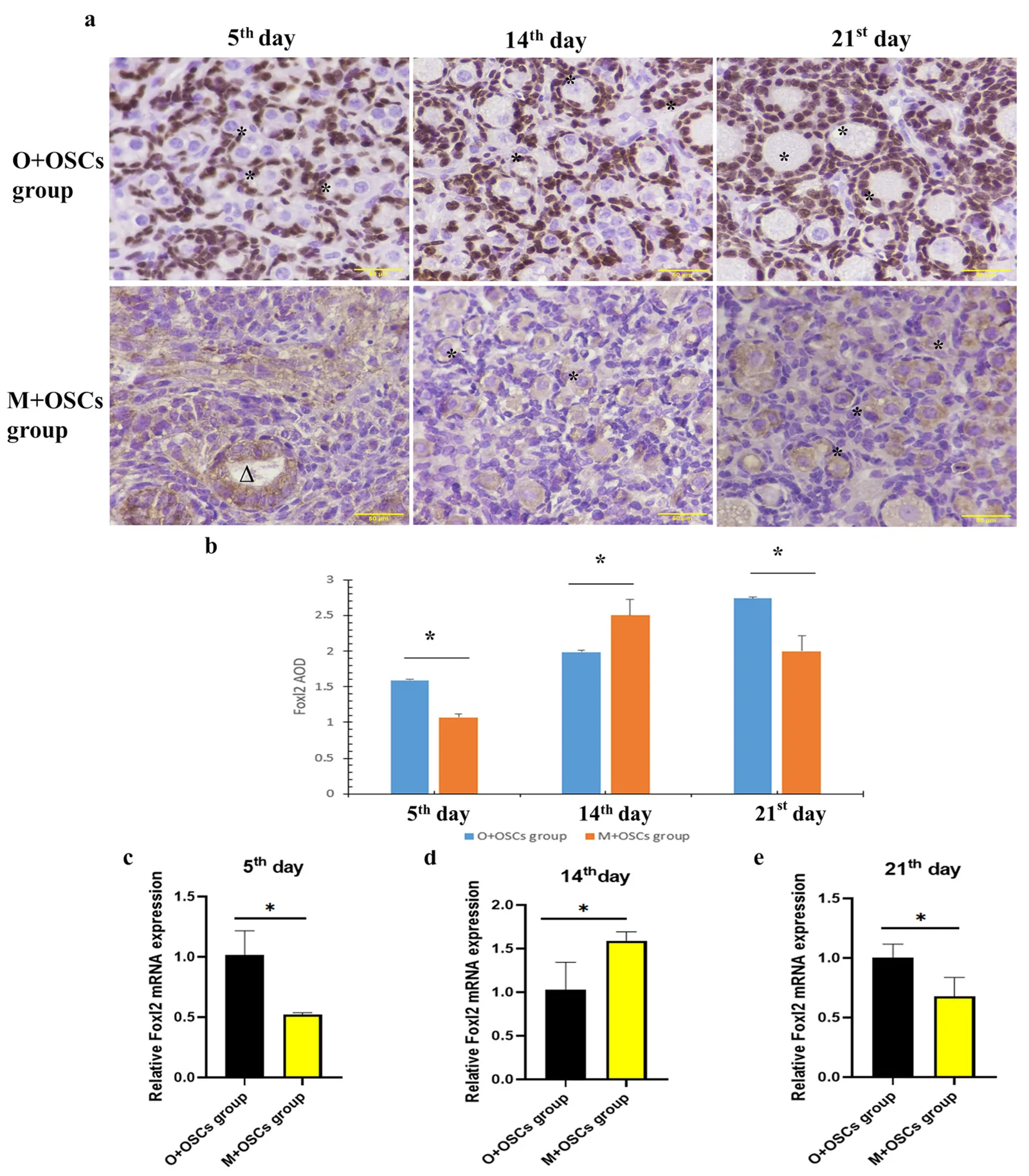
Immunohistochemistry and mRNA expression results of Foxl2 in co-cultured cell clusters. (**a**) Foxl2 localization with semi-quantitative results after hematoxylin re-staining (50 μm). * represents follicle-like structures, Δ represents mesonephric duct. (**b**) shows the expression of Foxl2 in the two groups at different co-culture times, and (**c**–**e**) show the results of Foxl2 mRNA expression in the two groups. Data are presented as mean ± SD (*n* = 3 independent experiments). Statistical significance was determined by one-way ANOVA followed by LSD post hoc test or unpaired two-tailed Student’s *t*-test, *p* < 0.05.

To further examine the molecular characteristics of granulosa and theca progenitor cells during in vitro follicle-like structure development in different co-culture systems, we systematically detected the spatiotemporal expression patterns of classical granulosa cell subtype markers (Foxl2 and Lgr5) and the theca progenitor cell marker (Gli1) via immunohistochemistry (IHC), immunofluorescence (IF), and quantitative real-time PCR (qRT-PCR). Foxl2 and Lgr5 are well-recognized specific markers that define two distinct subtypes of mouse ovarian granulosa cells and are essential for judging granulosa cell differentiation and maturation. IHC staining results revealed distinct temporal expression trends of Foxl2 between the two groups. In the O + OSC group, Foxl2 protein expression exhibited a continuous and significant upregulation throughout the entire co-culture period (*p* < 0.05). In contrast, Foxl2 expression in the M + OSC group displayed a transient peak at day 14 of co-culture. Statistical analysis further confirmed that Foxl2 protein levels were significantly higher in the O + OSC group than in the M + OSC group at day 21 (*p* < 0.05) (Figure 3a,b). Consistent with the protein-level changes, qRT-PCR validation demonstrated that Foxl2 mRNA expression in the M + OSC group peaked at day 14 and subsequently decreased significantly at day 21 (*p* < 0.05) (Figure 3c).

**Figure 4:**
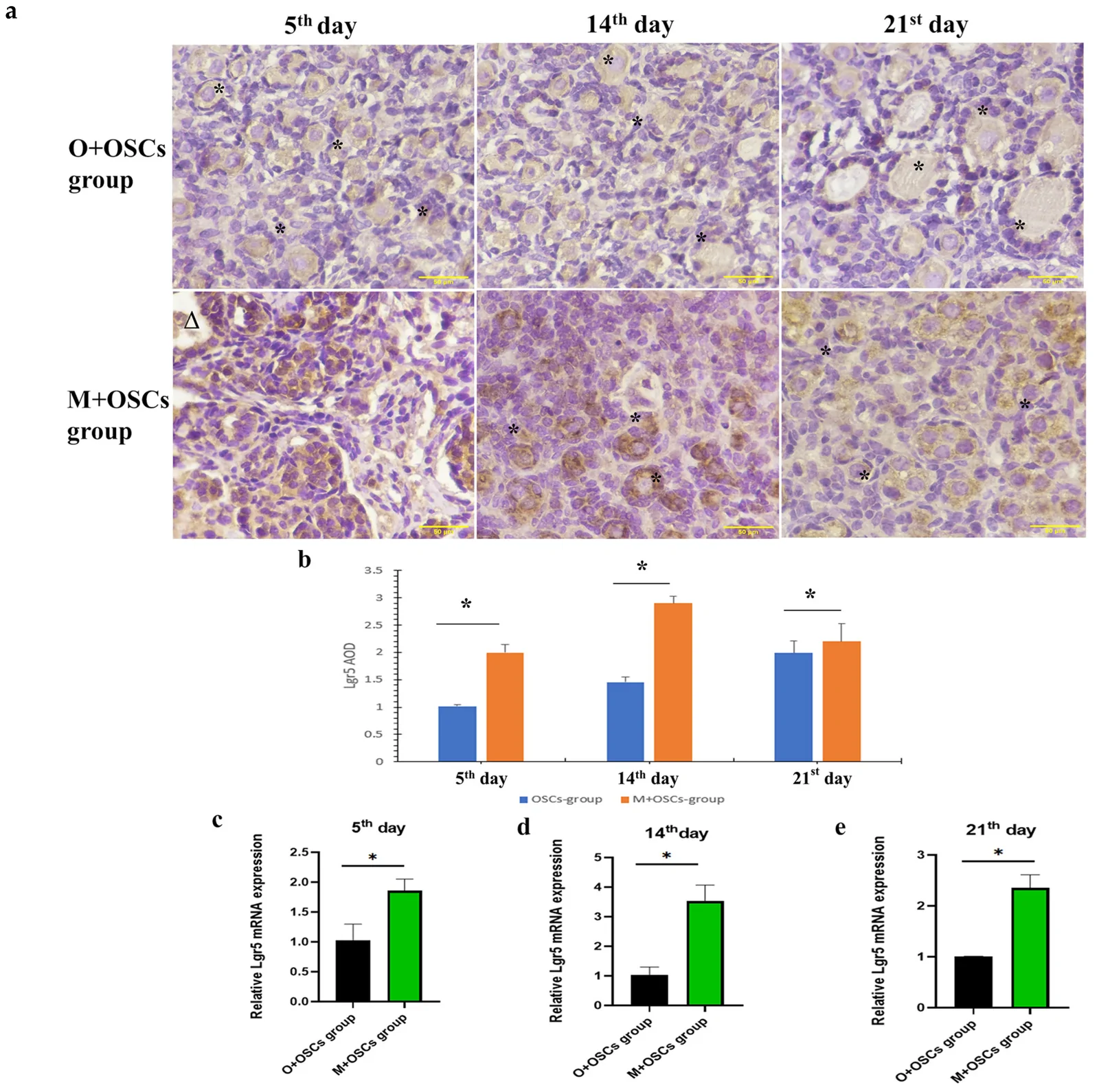
Immunohistochemistry and mRNA expression results of Lgr5 in co-cultured cell clusters. (**a**) Lgr5 localization with semi-quantitative results after hematoxylin re-staining (50 μm). * represents follicle-like structures, Δ represents mesonephric ducts. (**b**) shows the expression of Lgr5 in the two groups at different co-culture times, and (**c**–**e**) show the results of Lgr5 mRNA expression in the two groups. Data are presented as mean ± SD (*n* = 3 independent experiments). Statistical significance was determined by one-way ANOVA followed by LSD post hoc test or unpaired two-tailed Student’s *t*-test, *p* < 0.05.

Subsequent detection of the Lgr5-positive granulosa cell subtype showed prominent group differences in Lgr5 expression. Overall, the M + OSC group exhibited substantially higher Lgr5 protein expression than the O + OSC group during co-culture. Quantitative analysis of the positive staining area indicated that the proportion of Lgr5-positive regions in the M + OSC group was significantly elevated at day 5 (*p* < 0.05), reached the highest level at day 14 (*p* < 0.05), and underwent a slight decline at day 21. Notably, although Lgr5 expression remained relatively higher in the M + OSC group at day 21, the intergroup difference no longer reached statistical significance (Figure 4a,b). qRT-PCR analysis further corroborated these IHC observations, verifying that Lgr5 mRNA expression in the M + OSC group was at a maximum at day 14 with significant statistical differences (*p* < 0.05) (Figure 4c).

**Figure 5:**
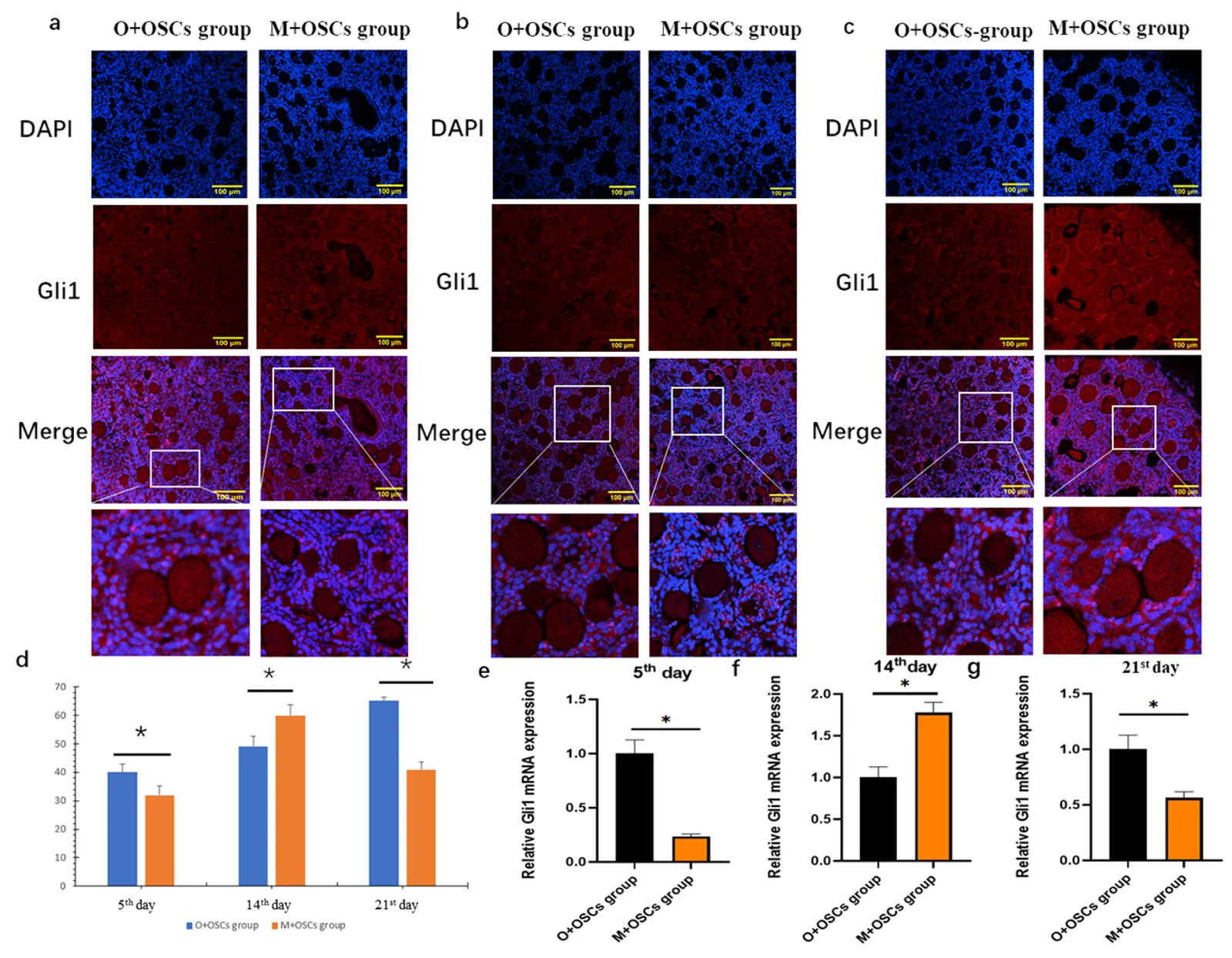
Immunofluorescence detection and mRNA expression of Gli1. (**a**–**d**) The expression of Gli1 fluorescent protein in the two groups on the 5th, 14th, and 21st days of co-culture. (**e**–**g**) Analysis of the expression of Gli1 mRNA in the two groups on the 5th, 14th, and 21st days of co-culture, respectively. * indicates significant differences between groups. Data are presented as mean ± SD (*n* = 3 independent experiments). Statistical significance was determined by one-way ANOVA followed by LSD post hoc test or unpaired two-tailed Student’s *t*-test, *p* < 0.05.

Given that mesonephros-derived ovarian theca progenitor cells specifically express Gli1 and stably persist in postnatal ovarian tissues, we further compared the dynamic expression of Gli1 to evaluate the differentiation status of theca progenitor cells in the two co-culture systems. IF staining analysis showed that Gli1 fluorescence intensity was higher in the O + OSC group than in the M + OSC group at day 5 and day 21. However, a reversed expression pattern was observed at day 14, where the M + OSC group displayed significantly stronger Gli1 fluorescence signals relative to the O + OSC group (*p* < 0.05). In terms of temporal dynamics, Gli1 expression in the O + OSC group increased steadily in a time-dependent manner during co-culture. By comparison, Gli1 expression in the M + OSC group peaked sharply at day 14 and declined at day 21, yet remained higher than its baseline level at day 5 (Figure 5a–d). qRT-PCR results were highly consistent with the IF data, confirming significantly higher Gli1 mRNA expression in the M + OSC group at day 14 (*p* < 0.05), whereas no remarkable intergroup differences were detected at day 5 and day 21 (Figure 5e–g).

### 2.4. Single-Cell Transcriptome Sequencing Identifies Distinct Cell Subsets in Co-Cultured Clusters

**Figure 6:**
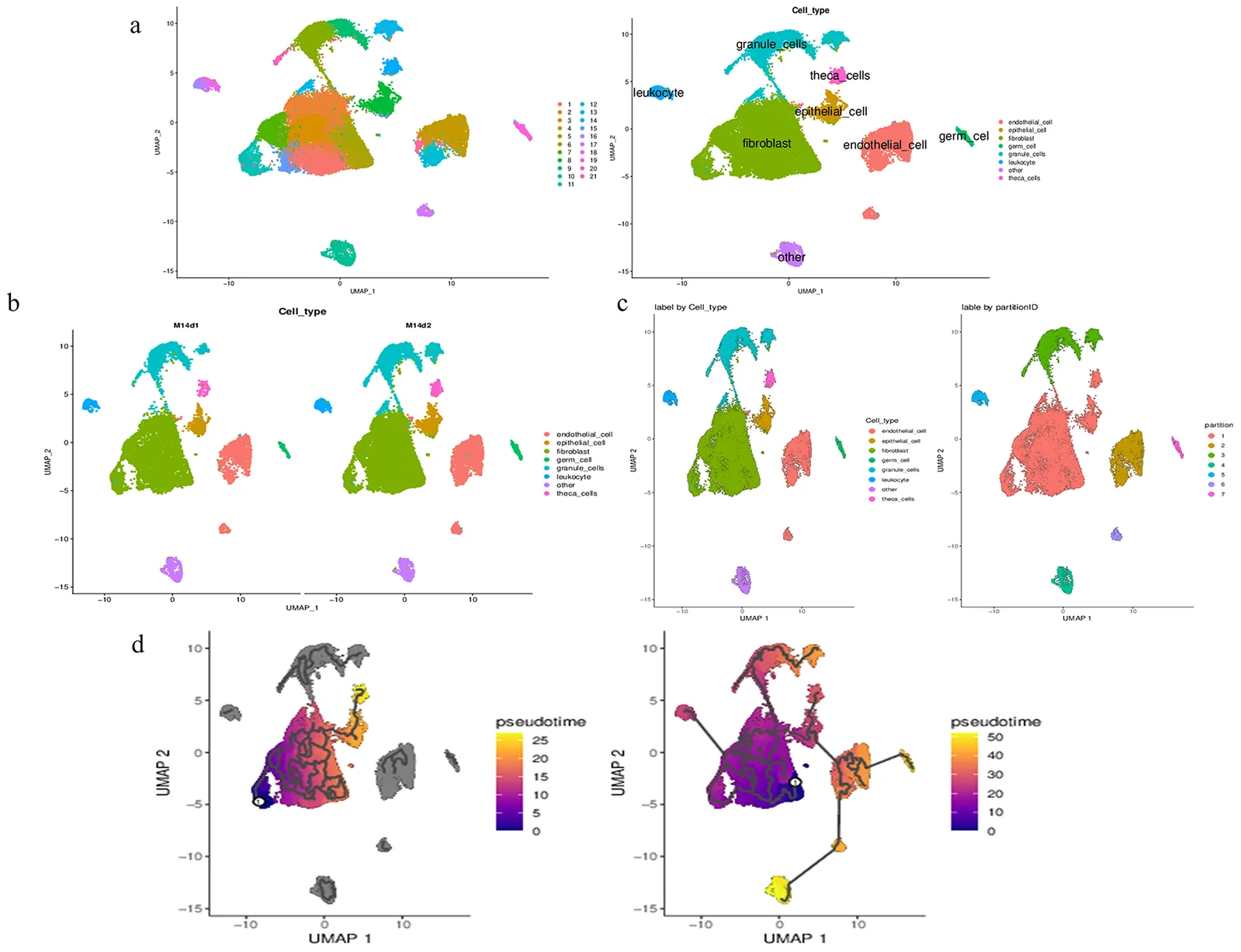
Cell clustering results of scRNA−seq. (**a**) Overall UMAP cell subgroup map. (**b**) UMAP cell subgroup maps of two biological replicates of the M + OSC group, showing consistent cell–type distributions. (**c**) Proposed time distribution map. (**d**) Cluster trajectory chart. scRNA−seq data were analyzed using Cell Ranger (v.6.1.2) and Seurat. Cell clustering was performed using the Louvain algorithm. Differential expression was determined using the DECENT software package (version 1.0.0) with a likelihood ratio test; *p* < 0.05 was considered statistically significant.

To systematically resolve the cellular composition and heterogeneity of follicle-like structure clusters derived from mesonephros and OSC co-culture, we performed 10× Genomics single-cell RNA sequencing on co-cultured tissues harvested at day 14, the optimal time point for follicle-like structure development observed in preceding experiments. A total of 11,075 high-quality single-cell transcriptomes were successfully captured and analyzed. Based on unbiased gene expression profiling and cell clustering, eight distinct cell subsets were identified within the co-cultured system, including endothelial cells, epithelial cells, stromal fibroblasts, germ cells, granulosa cells, leukocytes, and theca cells (Figure 6a). The proportional distribution of each cell type across biological replicates was visualized and confirmed to be consistent (Figure 6b,c). Among all identified populations, stromal fibroblasts, as the primary cellular component of ovarian stromal tissue, constituted the largest proportion. Importantly, granulosa cells and endothelial cells, two core cell types supporting follicular growth and maturation, were also abundantly enriched in the co-cultured cell clusters (Figure 6b–d).

**Figure 7:**
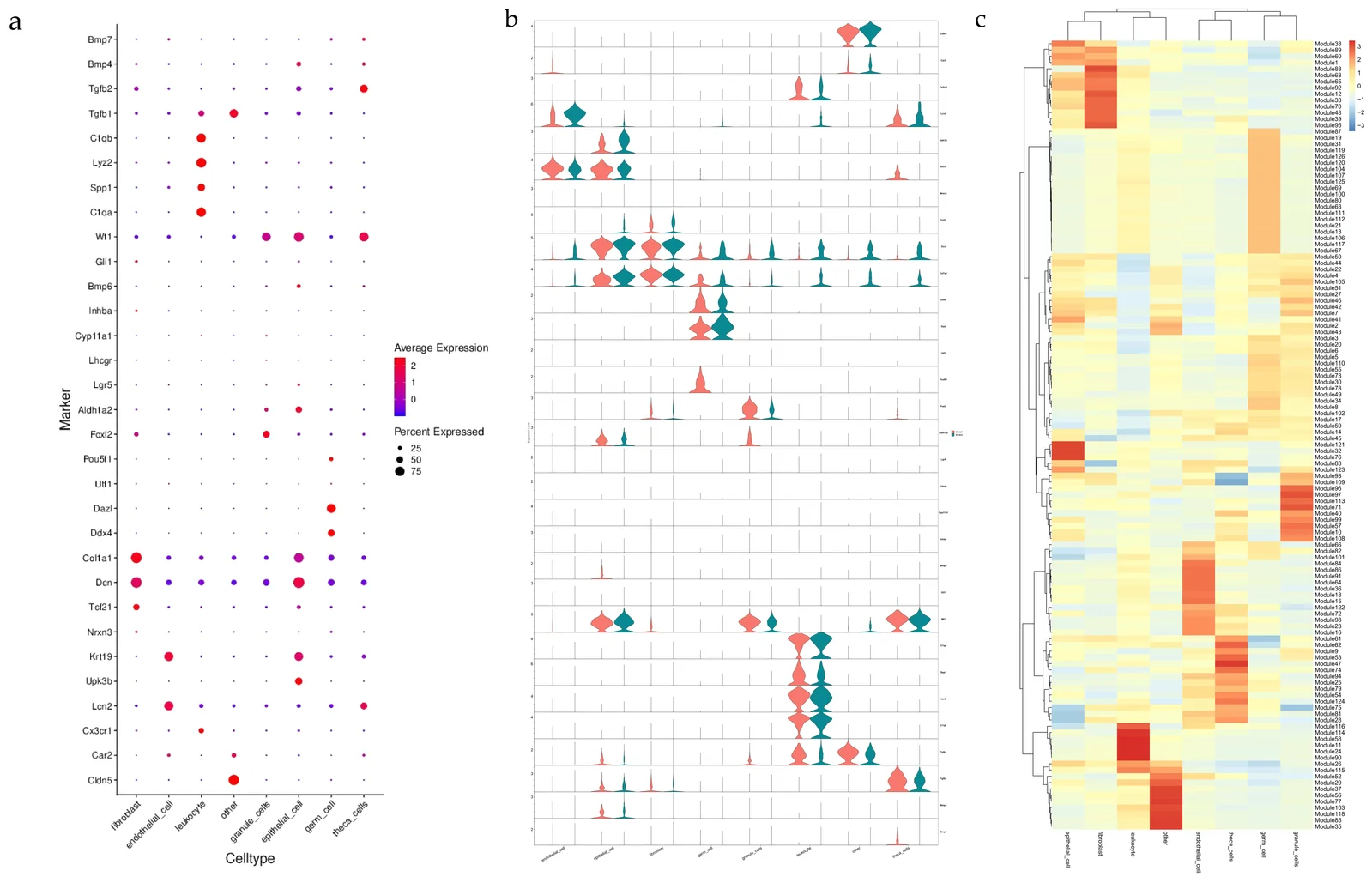
Results of differential gene expression in scRNA−seq. (**a**) Dot plot of marker genes. (**b**) Violin plot of marker genes. (**c**) Heatmap of gene co–expression modules. Differential expression analysis was performed using the DECENT software package with a likelihood ratio test. A *p*-value < 0.05 was considered statistically significant.

To verify the identity and molecular characteristics of each cell subset, we mapped the expression of canonical cell-specific marker genes across all clusters. Cell-type annotation was strictly validated using well-characterized marker signatures. Specifically, specific expression of Foxl2 and Lgr5 was detected exclusively in the granulosa cell cluster, further confirming the existence of two phenotypically distinct granulosa cell subtypes. Meanwhile, the dot plot visualization of the top 5–10 representative marker genes for each cell population further verified the specific enrichment of Foxl2 and Lgr5 in granulosa cells and Gli1 in theca cells at the single-cell level (Figure 7a,b). Additionally, the theca cell subset exhibited specific Gli1 expression, which is a definitive marker of mesonephros-derived theca progenitor cells. These molecular profiling results provide robust cellular evidence that mesonephros-derived cell populations participate in and facilitate in vitro follicle-like structure formation (Figure 7a,c).

### 2.5. Transcriptome Analysis Reveals Enrichment of the PI3K/AKT Pathway and Differentially Expressed Genes

**Figure 8:**
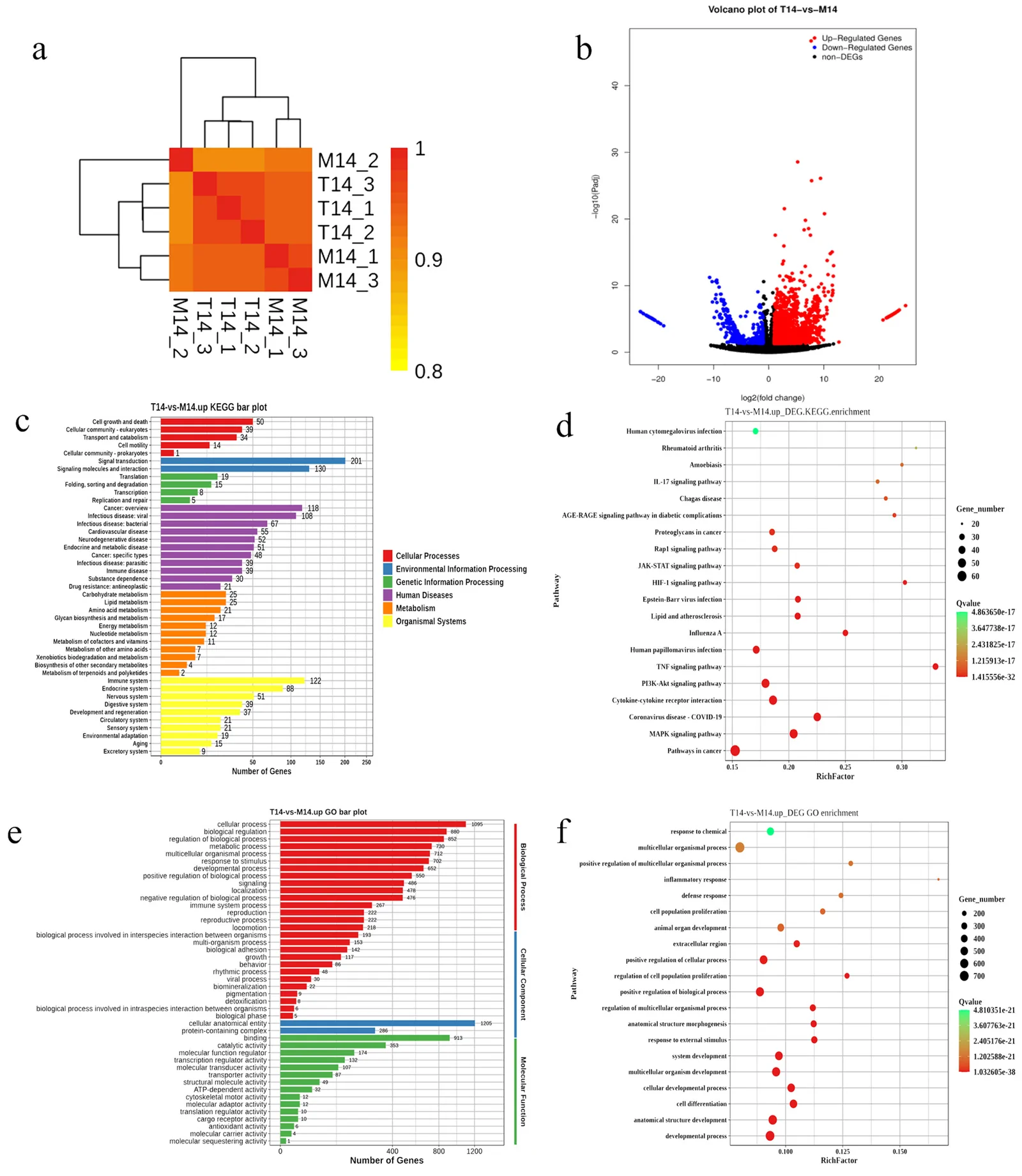
Analysis results of RNA sequencing data in co–culture cell clusters. (**a**) Heatmap of the results of correlation analysis of the same group of samples. (**b**) Volcano map of gene differential expression in different subgroups. (**c**) KEGG distribution histogram of different groups of differential genes. (**d**) KEGG–rich distribution map of differentially expressed genes. (**e**) Differential GO distribution histogram. (**f**) Differentially expressed GO–rich distribution point diagram. T14–1, T14–2, and T14–3 represent three repeat samples of O + OSC group co–culture at 14 days, and M14–1, M14–2, and M14–3 represent three repeat samples of M + OSC group co–culture at 14 days. Differential expression analysis was performed using DESeq2 (v.1.22.2) and edgeR (v.3.6.8) with a threshold of FDR < 0.05 and |log2FC| > 1. Functional enrichment analysis was conducted using KEGG and GO databases.

To elucidate the global transcriptomic alterations and core signaling mechanisms by which mesonephros cells regulate OSC-derived follicle-like structure development, we performed bulk RNA sequencing using day 14 co-cultured samples from the experimental M + OSC group and the control O + OSC group. Intragroup correlation analysis revealed high transcriptomic consistency among biological replicates, demonstrating excellent sample reproducibility and reliable grouping design for subsequent differential analysis (Figure 8a). Differential gene expression analysis identified a total of 2147 differentially expressed genes (DEGs) between the two groups, consisting of 1961 significantly upregulated genes and 186 downregulated genes in the M + OSC experimental group (Figure 8b). Functional enrichment analysis revealed that the PI3K/AKT signaling pathway was prominently and significantly enriched in the M + OSC group, implying its potential central role in mediating mesonephros-induced follicular differentiation (Figure 8c,d). Gene Ontology (GO) functional annotation further demonstrated that the DEGs enriched in the experimental group were primarily involved in the regulation of multicellular biological processes, the positive modulation of cellular and biological processes, multicellular organism development, and anatomical structure morphogenesis, as well as cell differentiation and developmental processes. Consistently, the functional terms associated with upregulated DEGs were predominantly enriched in cell proliferation, embryonic organ development, proliferative regulation, structural morphogenesis, and cell fate differentiation, which collectively support the pro-development and pro-differentiation effects of mesonephros cells on folliculogenesis (Figure 8e,f).

### 2.6. Upregulation of AKT and VEGF Is Confirmed by Western Blotting, IHC, and qRT-PCR

**Figure 9:**
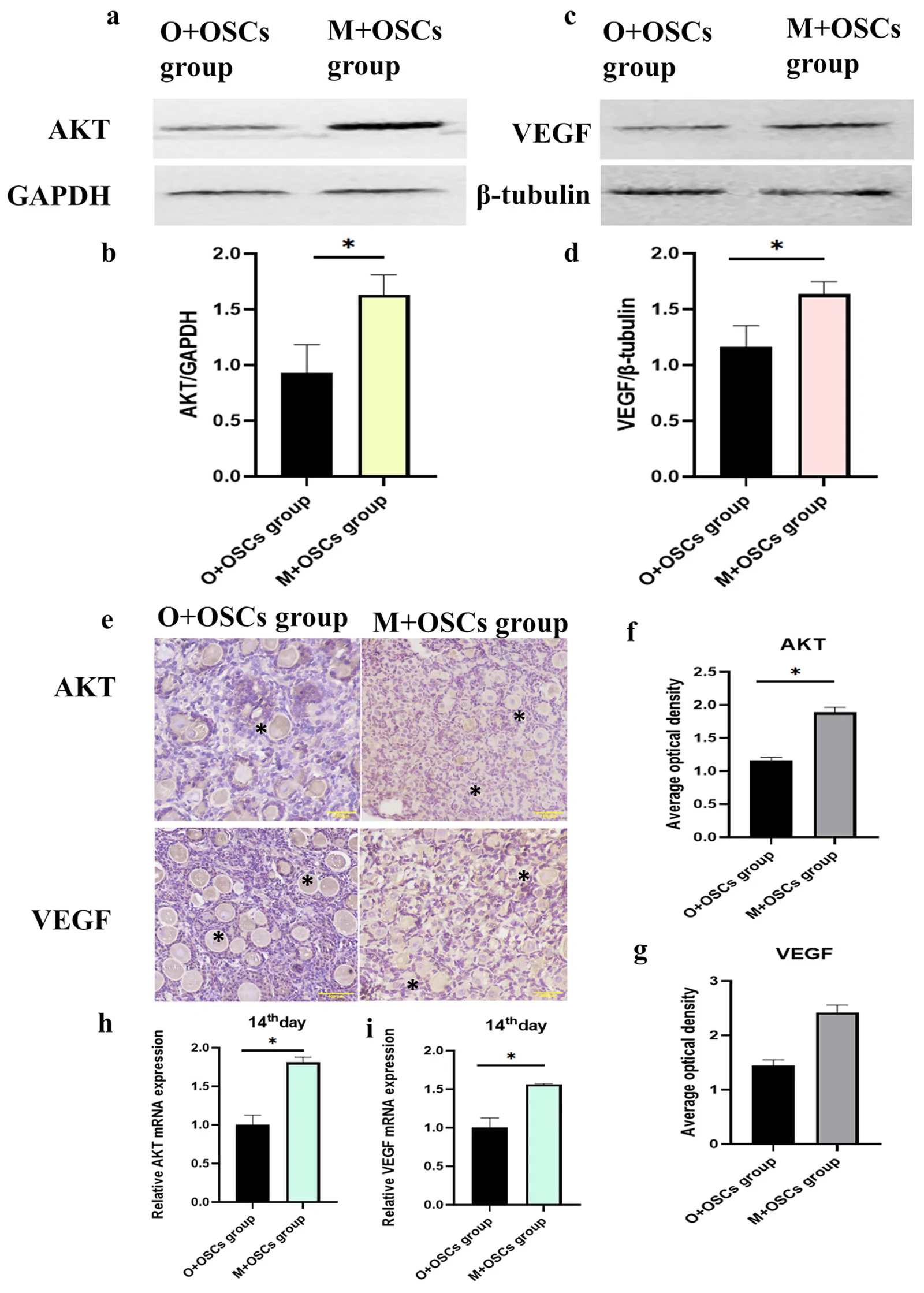
AKT/VEGF protein and mRNA expression were detected on the 14th day of co-culture. (**a**–**d**) Protein expression results of AKT and VEGF. The figure shows the cropped blot image. (**e**–**g**) AKT/VEGF localization with semi-quantitative results after hematoxylin re-staining (100 μm). * represents follicle-like structures. (**h**,**i**) show the results of AKT/VEGF mRNA expression in the two groups. Data are presented as mean ± SD (*n* = 3 independent experiments). Statistical significance was determined by unpaired two-tailed Student’s *t*-test, *p* < 0.05.

Given the significant enrichment of the PI3K/AKT signaling pathway revealed by transcriptomic sequencing, we further validated the key functional genes within this pathway to confirm the gene set enrichment status during mesonephros-mediated follicle-like structure development. We focused on two pivotal co-expressed genes, AKT and VEGF, and verified their expression patterns at the protein and mRNA levels via Western blotting, immunohistochemistry (IHC), and qRT-PCR. Western blotting results showed that the protein levels of AKT and VEGF were markedly and significantly elevated in the M + OSC group compared with the O + OSC control group (*p* < 0.05) (Figure 9a–d). Consistent with the protein quantification results, IHC staining further validated the stronger positive immunoreactivity of AKT and VEGF in the M + OSC co-cultured clusters, while the O + OSC group displayed relatively weak staining signals, with significant intergroup differences (*p* < 0.05) (Figure 9e–g). Furthermore, qRT-PCR analysis confirmed that the mRNA transcription levels of AKT and VEGF were substantially upregulated in the M + OSC group relative to the control group (*p* < 0.05) (Figure 9h,i). Collectively, these multi-level validation results solidly confirm the enrichment of the PI3K/AKT signaling pathway in the mesonephros-dependent follicle-like structure differentiation system.

### 2.7. Morphological and Functional Assessment of Co-Cultured Tissues After Renal Subcapsular Transplantation

**Figure 10:**
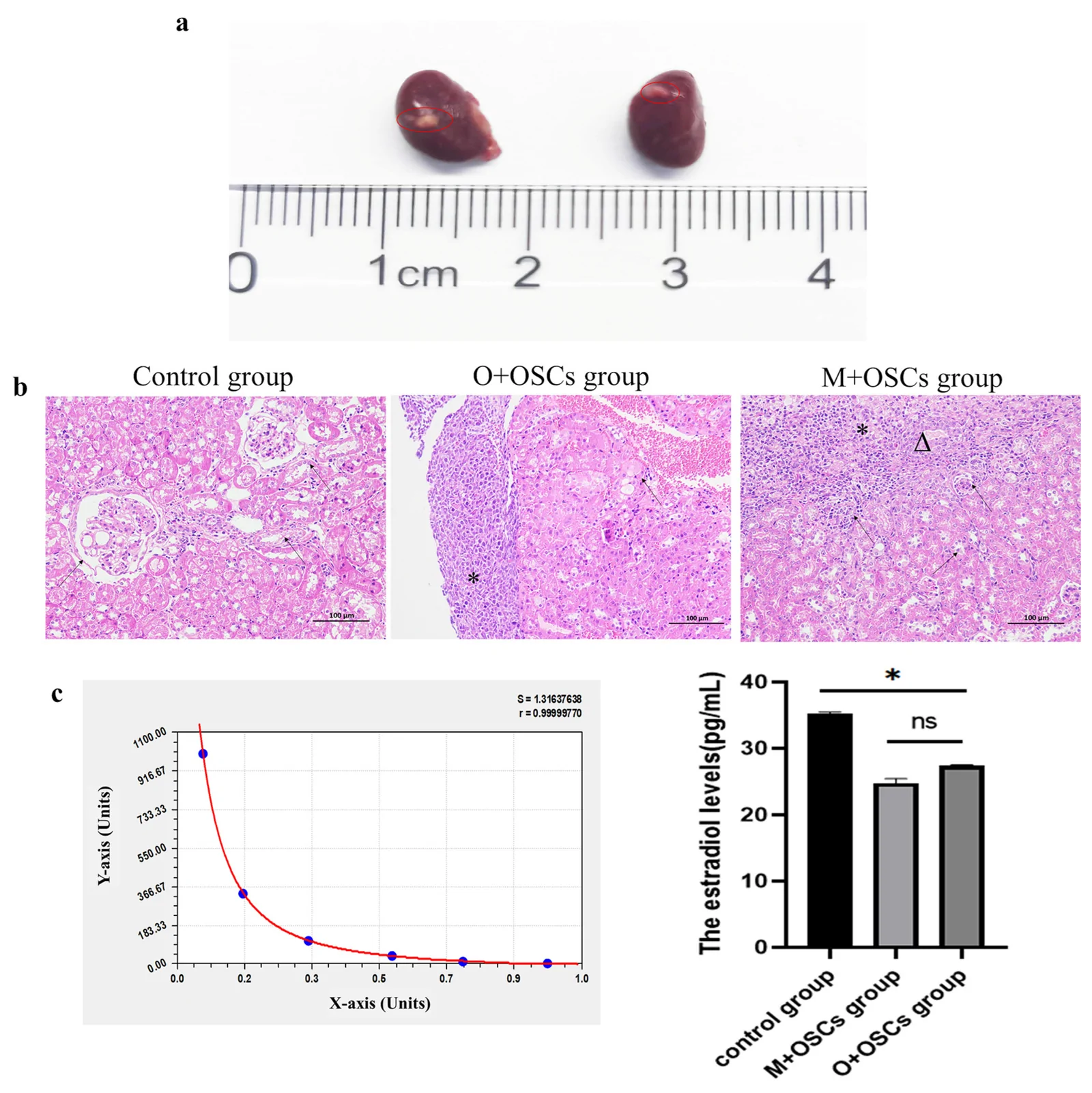
HE staining results of kidney subcapsular transplantation of co-cultured tissue and hormone testing (100 μm). (**a**) Co-culture tissue and kidney tissue 21 days after renal subcapsular transplantation. The part of the implanted tissue is circled in red. (**b**) HE staining results of paraffin sections of renal subcapsular transplantation in control group, M + OSC group, and O + OSC group. Arrow points to the glomeruli and renal tubules; * represents follicle-like structures; Δ represents mesonephric ducts. (**c**) The results of whole-blood estradiol level on the 21st day after renal subcapsular transplantation. Data are presented as mean ± SD (*n* = 3 mice per group). Statistical significance was determined by one-way ANOVA followed by LSD post hoc test, *p* < 0.05.

To evaluate the in vivo developmental potential of co-cultured cell clusters, we transplanted tissues from the M + OSC and O + OSC groups under the renal capsule of recipient mice. On day 21 post-transplantation, the grafted tissues were clearly visible under the renal capsule (Figure 10a). Morphologically and structurally, the M + OSC group showed similarities to the O + OSC group, with no significant differences observed (Figure 10b).

We further quantified serum estradiol (E2) levels using ELISA on day 21 post-transplantation. Compared with the control group (non-transplanted or sham-operated), both the M + OSC and O + OSC groups exhibited significantly lower E2 secretion levels (*p* < 0.05). Specifically, the E2 level in the M + OSC group was significantly lower than that in the control group. Although the E2 level in the M + OSC group was lower than that in the O + OSC group, the difference between the two co-culture groups was not statistically significant (Figure 10c).

## 3. Discussion

As a transient organ during fetal development, the contributions of the mesonephros to the growth and development of female gonads have been rarely documented. Previous studies have hypothesized that the mesonephros is closely associated with the first wave of folliculogenesis in the ovary [27] and may serve as an important source of ovarian surface epithelial progenitor cells [28]. A significant number of mesonephros-specific genes are expressed in ovarian surface epithelial progenitor cells [29], suggesting that the mesonephros not only influences fetal kidney development but also plays a crucial role in the genesis and differentiation of follicles. With the introduction of the concept of OSCs, we have established a more refined model for investigating the development and differentiation of follicles during the embryonic stage. While there have been reports on the isolation, establishment, and in vitro differentiation of OSCs [30], research on inter-organ interactions during OSC differentiation remains scarce. Given that the mesonephros is present during the early stages of embryonic development [31] and has multifaceted relationships with follicular genesis and differentiation [32], our study seeks to validate the involvement of the mesonephros in the genesis and development of follicles and granulosa cells by facilitating the differentiation of oogonial stem cells. As expected, our study demonstrates for the first time that the mesonephros contributes to OSC differentiation in vitro, supporting the multiple reports that the mesonephros is an important source of pregranulosa cells and follicular theca progenitor cells.

Firstly, we observed the follicle-like structure formation in co-cultured clusters of mesonephros cells and OSCs through morphological observations. The HE staining results revealed that, by the 14th day of co-culture, a substantial number of follicle-like structures could be observed under the microscope. Although the maturity of these follicle-like structures was not as high as that in the O + OSC group, their number was significantly greater. However, by the 21st day of co-culture, the number of follicle-like structures in the mesonephros cell and OSC co-culture group had significantly decreased. This indicated that mesonephric action was markedly diminished in the late co-culture period, mimicking in vivo development.

The results of live-cell workstation tracer observations and follicle counting showed that no follicle-like structures were formed during the initial days of co-culture between mesonephros cells and OSCs, with the morphological structure of the co-cultured cells resembling that of the ovarian interstitial part. In the early stages of fetal ovarian development, mesonephros cells first appear in the ovarian medulla [33], and by the 8th week of embryonic development, the mesonephric tubules gradually degenerate into fragments, primarily existing in the ovarian stroma [34]. This suggests that the addition of mesonephros cells stimulates the development of dormant primordial follicles in OSCs during normal reproductive cycles simulated in vitro. Through careful observation of cell migration and fusion using the live-cell workstation, it was found that the number of newly formed follicle-like structures in the M + OSC group peaked on the 14th day of co-culture and gradually decreased thereafter, which may be attributable to the gradual decline of the mesonephros over time. The statistical results of follicle counting from HE staining also supported this observation. Therefore, in this experimental series, we concluded that the optimal time for the mesonephros to promote OSC differentiation and follicle-like structure formation was on 14 days of co-culturing.

The ovarian basement membrane is a key factor influencing the proliferation and differentiation of granulosa cells [35]. Evidence suggests that stem cell models can induce the production of parietal granulosa cells and cumulus granulosa cells, both of which originate from pregranulosa cells [36]. The contribution of the mesonephros to follicle-like structure development may be closely related to the development of granulosa cells. Granulosa cells can be divided into two groups, one labeled with Foxl2-positive cells and the other labeled with Lgr5-positive cells. Lgr5-labeled granulosa cells can maintain female reproductive function for a long time and gradually transform into Foxl2-positive cells, thereby participating in the formation of primordial follicles. The IHC results showed that, on the 5th day of co-culture, the positive expression of Foxl2 in the O + OSC group was significantly higher than that in the M + OSC group. However, a considerable number of primordial follicle-like structures and their surrounding monolayer granulosa cells were observed in the M + OSC group. On the 14th day of co-culture, the positive expression area of Foxl2 in the M + OSC group was significantly higher than that in the OSC group, suggesting that the primary role of the mesonephros in the early differentiation of OSCs is activating dormant primordial follicles. During the middle stage, it appears to play a more substantial role in stimulating the growth and development of oocytes, leading to a significant increase in follicle-like structure development. This may indicate the initiation of the first wave of follicle development and the formation of the initial cohort of granulosa cells [37]. On the 21st day of co-culture, the positive expression of Foxl2 in the M + OSC group was significantly reduced. This decline may be related to the gradual regression and functional transformation of the mesonephros over time. Meanwhile, the differentiation and maturation of OSCs continued, promoting follicle development under the regulation of neurohumoral factors [38]. The expression trend of Foxl2 mRNA further confirmed the IHC results, demonstrating that the mesonephros played a significant role in promoting granulosa cell proliferation in follicles. Unlike Foxl2, the expression pattern of Lgr5 showed a high expression trend in the M + OSC group and was observed on the 5th day of co-culture, indicating a close relationship between Lgr5-positive cells in the ovarian epithelium and the mesonephros. The mRNA expression of Lgr5 further confirmed the IHC findings and supported the promotion of the development and proliferation of two groups of granulosa cells by the mesonephros.

On the 14th day of co-culture, the expression levels of Gli1 fluorescent protein and its mRNA reached their peak in the M + OSC group. According to the follicle-like structure count results, the highest number of follicle-like structures was observed on the 14th day of co-culture between mesonephros cells and OSCs. This phenomenon provided the basis for the highest levels of Gli1 protein expression in the co-culture group of mesonephros cells and OSCs on the 14th day. Although the number of follicle-like structures was low on the 5th day of co-culture, Gli1 protein was still expressed at this time, suggesting that cells derived from the mesonephros not only participated in the first wave of follicle development but were also involved in the formation of pregranulosa cells in vitro. With the extension of co-culture time, the expression of Gli1 protein gradually increased in the O + OSC group, indicating that Gli1-labeled positive cells in the O + OSC group played different roles compared to Gli1 cells from the mesonephric origin. Mesonephros-derived cells made significant contributions to the in vitro development of follicles and granulosa cells.

Based on the above experimental results, we inferred that the 14th day of in vitro co-culture is the optimal time for the action of the mesonephros. Therefore, we sequenced the cell clusters formed on the 14th day of co-culture between mesonephros cells and OSCs. Following identification through 10 × Genomics single cell transcriptome sequencing, and combined with multi-sample integration analysis and different cell types, the cluster analysis showed that the samples could be divided into 21 cell subgroups. Based on trajectory clustering, these subgroups were further integrated into eight cell clusters. Among these, the granulosa cell group accounted for 17.02%, while the theca cell group accounted for 2.17%. This result further confirms the existence of follicles of mesonephric origin, which are closely related to the occurrence of granulosa cells. In the dot plot of gene expression markers, granulosa cells specifically expressed Foxl2 and Lgr5, as well as mesonephros theca cell-enriched genes such as Lhcgr-, Cyp11a1-, Aldh1a1-, and Gli1-positive cells from the mesonephros [39]. Collectively, the cell subsets and their specific expression genes present in the co-cultured cell clusters of mesonephros cells and OSCs bear a strong resemblance to the cell types found in the ovary.

Transcriptome sequencing revealed genetic changes associated with follicle-like structure formation and differentiation in the co-cultured cell clusters of mesonephros cells and OSCs. The results indicated that Gli1 was significantly upregulated in the co-cultured cell clusters, providing further direct evidence of the involvement of the mesonephros in follicle and granulosa cell formation. Gli1-positive cells in the mesonephros first appear in the medulla of neonatal ovaries and participate in the development of the first wave of follicles [40]. The progenitor cells of the ovarian theca initially acquire a positive marker for the specific expression of Gli1 in response to the paracrine signals of Desert hedgehog and Indian hedgehog from granulosa cells [41]. In the absence of these two signaling pathways, ovarian tissue exhibits a loss of its theca layer, restricting steroid secretion, severely impairing follicle development, and preventing the further formation of the corpus luteum [42,43]. Two days after birth, the expression of Gli1 spreads into the ovarian stroma, and Gli1-positive cells are present in the stroma surrounding Foxl2-positive granulosa cells, which persist in adult ovaries [44,45]. Further research is needed to elucidate specific regulatory mechanisms, such as the regulation of multicellular biological processes, positive regulation of cellular processes, positive regulation of biological processes, and regulatory mechanisms of multicellular biological development. Nevertheless, the sequencing results suggest that there is significant enrichment of the PI3K/AKT signaling pathway.

According to the transcriptome sequencing results, compared with the control group, the most important biochemical metabolic pathways and signal transduction pathways involved in differentially expressed genes were identified in the mesonephros cell and OSC co-culture groups through significant pathway enrichment. Among them, the PI3K/AKT signaling pathway is one of the top 20 most significantly enriched pathways. In order to verify the role of the PI3K/AKT signaling pathway in promoting OSC differentiation by the mesonephros, we detected the important signaling molecules AKT and VEGF. The simultaneous detection of these two factors is justified by the feedback and cross-talk mechanism between the VEGF/VEGFR2 and the PI3K/AKT signaling pathways—that is, after VEGF is activated by VEGFR2, it can indirectly activate AKT. Conversely, AKT, under certain conditions, can regulate VEGF expression through transcription factors such as HIF-1α, thereby forming a potential positive feedback loop. Thus, VEGF and AKT exert a synergistic effect. Western blot analysis showed that the expression levels of AKT and VEGF were significantly increased in the mesonephros cell and OSC co-culture groups. Meanwhile, immunohistochemical detection and real-time PCR further verified the protein expression results. We verified the upregulation of PI3K/AKT signaling pathway genes through different perspectives. The results showed that the biological process of the mesonephros promoting OSC differentiation into follicles in vitro was closely related to the PI3K/AKT signaling pathway. It should be noted that our research mainly relies on multi-gene transcriptional signatures and KEGG enrichment, rather than on the determination of phosphorylation activity to demonstrate that the PI3K/AKT signaling pathway is involved in the promotion of the differentiation of OSCs in vitro by the mesonephros. While this method has certain limitations, our reconstruction ensured that our evidence is consistent with the scope of the research hypothesis. The convergence of KEGG pathway enrichment, the upregulation of core genes, and changes in total protein points to the PI3K-AKT signaling pathway as an important candidate pathway. In the subsequent research, we will conduct further targeted functional validation.

To further validate the physiological function of mesonephros-derived cells in follicle-like structure formation, we used cell clusters formed by co-culturing mesonephros and OSC for 14 days and performed ovarian castration and renal subcapsular transplantation experiments in severely immunodeficient mice. Morphological observations using HE staining revealed no local adhesion or rejection reactions in the kidney tissue after implantation of the in vitro co-culture tissue. After 21 days of transplantation, new follicle-like structures appeared under the renal capsule, but their volume was small and their quantity limited, likely due to mechanical interference during the transplantation process. Potential reasons for the limited follicle-like structure growth include mechanical damage to co-cultured cell clusters with short-term inflammatory reactions. Hormone level measurements 21 days post-surgery showed that the E2 levels in the M + OSC group were lower than those in the normal control group, but not significantly different from the O + OSC group. These findings suggest that follicle-like structures derived from the mesonephros cells may possess certain estrogen secretion capabilities, though their effectiveness was limited. Although co-cultured cell clusters exhibited good growth status under the renal capsule after 21 days of in vivo growth, further observation and research are required to fully restore their physiological functions and maximize their functional roles.

Several limitations of this study should be acknowledged. Our findings are primarily derived from an in vitro co-culture system, which cannot fully replicate the complex in vivo environment. Additionally, the absence of direct phospho-protein measurements means that our conclusions regarding PI3K/AKT pathway activation remain correlative rather than causal. The transplantation experiment showed only partial functional recovery, possibly due to the short observation period. It should be noted that endocrine function was assessed primarily according to E2 levels, and a more comprehensive hormone panel will be necessary in future studies. Future studies incorporating pharmacological inhibitors or genetic knockdown approaches are needed to establish causality.

The embryonic development of follicles and granulosa cells plays a crucial role in the genetics and evolution of mammals. From a developmental perspective, it is important to determine the source of precursor cells for follicles and granulosa cells. Our study demonstrated the role of the mesonephros in OSC differentiation and follicle-like structure formation in vitro, identified the optimal time for the mesonephros to promote OSC differentiation, and studied the signaling pathways related to this process. Understanding the contribution of the mesonephros to follicle-like structure formation is crucial for studying early embryonic development and improving female fertility.

## 4. Materials and Methods

### 4.1. Ethics Statement

The experimental protocol was approved by the Inner Mongolia Medical University Medical Ethics Committee (YKD202404020). All animal handling methods were conducted in full compliance with the animal handling guidelines to ensure animal welfare. Clinical trial number: Not applicable.

### 4.2. Animals

In this study, E10.5 female fetal mice of the C57BL/6N-GFP, 6–8-week-old female SCID beige mice, and ICR female mice within 3 days of birth, provided by the Experimental Animal Center of Inner Mongolia University (Experimental Animal License Number: SCXK (Mongolia) 2020-0002), were maintained in an SPF-grade animal facility, with the temperature kept between 20 °C and 24 °C, and were kept on a natural circadian cycle, with free access to food and water. During the sample collection process, the mice were euthanized using a high-concentration carbon dioxide asphyxiation method.

### 4.3. Grouping

Ovary cell and OSC co-culture group (O + OSC group): Primary OSCs were extracted and co-cultured with ovarian somatic cells. Mesonephric cell and OSC co-culture group (M + OSC group): Primary cells of mesonephros were extracted and co-cultured with OSCs. According to the reproductive cycle of mice, we set the 5th, 14th, and 21st days of co-culture as the observation points.

For the transplantation experiment, there were three groups: (i) control group (non-surgical, intact mice); (ii) O + OSC group (ovariectomized mice receiving ovarian somatic cell + OSC co-cultured grafts); and (iii) M + OSC group (ovariectomized mice receiving mesonephros + OSC co-cultured grafts).

### 4.4. Materials from the Mesonephros

The embryo days of the mesonephros were calculated by detecting vaginal plugs. Female fetal mice were selected on the 10.5th day of embryonic development based on the morphology of their genital ridge: in female fetuses, the genital ridge appears as a slender, cord-like structure with a smooth surface and no prominent vascularization, clearly demarcated from the mesonephros; in contrast, male fetuses exhibit a relatively thicker genital ridge with visible vascular texture. Sex was further confirmed using the C57BL/6N-GFP transgenic mouse line. The genital ridge and mesonephros were separated using a 1 mL syringe needle.

Primary single cells were obtained from mesonephric tissue using a two-step enzymatic digestion method. Briefly, tissues were digested with 0.1% collagenase IV (Gibco, Waltham, MA, USA) in DPBS at 37 °C for 25 min, neutralized with serum-containing medium, and centrifuged at 1500 rpm for 5 min at 4 °C. The pellet was then digested with 0.05% trypsin (Gibco, USA) at 37 °C for 4 min, terminated with serum-containing medium, and filtered through a 40 μm cell strainer (Corning, Corning, NY, USA). Cell yield and viability were assessed through hemocytometer and trypan blue exclusion, yielding approximately 1–2 × 10^6^ cells per mesonephros pair with >90% viability. Our previous findings [14] showed that mesonephros-derived cells were characterized by fibroblast-like spindle morphology and specific marker gene expression prior to co-culture.

### 4.5. Extraction and Purification of OSCs

#### 4.5.1. Extraction of OSCs

Twenty 3-day-old female neonatal mice (3dpp) were euthanized through high-concentration CO_2_ asphyxiation, immersed in 75% alcohol for 3 min, and dissected under a stereomicroscope. Ovaries were isolated, cleaned of surrounding tissues in DPBS, and transferred to a 15 mL centrifuge tube containing 1 mL DPBS. After digestion at 37 °C with 5% CO_2_ for 15 min with gentle pipetting every 5 min, 1 mL FBS was added to terminate digestion, followed by centrifugation at 1500 rpm for 5 min. The pellet was then digested with 4 mL of 0.05% trypsin at 37 °C with 5% CO_2_ for 5 min, terminated with 1 mL FBS, and centrifuged again. The pellet was washed with 5 mL DPBS via repeated pipetting and centrifugation, and finally resuspended in OSC culture medium to obtain a single-cell suspension.

#### 4.5.2. Purification of OSCs

Next, 2 mL of OSC culture medium was added to resuspend the cells, and cells were inoculated into a 6-well cell culture plate at a density of 5.0 × 10^5^ cells per well. In the initial cultivation stage, all suspended cells were transferred to new wells at intervals of 45 min, 1 h and 45 min, and 4 h and 45 min. After overnight cultivation, OSCs grown in suspension as string clones were collected. After the suspended cells were transferred, the remaining adherent cells were further cultured to obtain ovarian somatic cells.

The OSCs used in this study were isolated and characterized according to the experimental method that we have previously established [14]. The OSCs extracted in this study have a typical circular or elliptical shape, grow in clusters like grapes, express reproductive cell markers including MVH, OCT-4, and Fragilis, and do not express ovarian somatic cell markers. Flow cytometry analysis confirmed the high purity (>90%) of the OSC population [14].

### 4.6. Co-Culture Program

Cell clustering was performed in 96-well U-bottom plates (Corning, USA). Mesonephros cells (or ovarian somatic cells) and OSCs were mixed at a 1:1 ratio (0.5 × 10^6^ cells each), resuspended at 6 μL per cluster, and incubated at 37 °C with 5% CO_2_. After 45 min, wells were filled with medium, and half of the medium was replaced after 8 h, followed by overnight culture. The 1:1 ratio was selected based on preliminary experiments showing optimal follicle-like structure formation with consistent morphology, whereas higher somatic cell proportions caused fibroblast overgrowth and lower proportions provided insufficient support. Quality control showed uniform clusters (176.3 ± 12.5 μm; CV = 7.1%; success rate > 95%). Clusters were then transferred onto the upper surface of a Transwell membrane (0.4 μm pore size, Corning, USA) with medium supplied from the lower chamber, maintaining an air–liquid interface for stable adhesion and nutrient access while allowing paracrine factor diffusion. Half of the medium was replaced daily.

### 4.7. HE Staining

Twenty co-cultured cell clusters were randomly selected from each group. On the 5th, 14th, and 21st days of co-culture, the tissues were fixed overnight at 4 °C using 4% paraformaldehyde (PFA). Subsequently, continuous sectioning was performed, and one slice was selected from every three for staining. The sections were stained with hematoxylin (Beyotime, Shanghai, China) for 1 min, followed by staining with eosin (Beyotime, China) for 30 s. After staining, the sections were sealed and observed under a microscope, where cells were counted and photographed.

### 4.8. Cell Migration and Follicle Count

On the 5th, 14th, and 21st days of co-culture, the migration of mesonephros cells carrying green fluorescent protein was observed in two groups of co-cultured cell clusters using a live-cell workstation (BioTek Cytation 5, Agilent, Santa Clara, CA, USA). By randomly selecting 20 co-cultured cell clusters from each group and calculating the number of follicles in five high-power fields per group, follicle counting was completed. Finally, statistical analysis was performed.

### 4.9. IHC Detection

The primary antibodies used were rabbit anti-Foxl2 monoclonal antibody (1:200, Abcam, Cambridge, UK), rabbit anti-Lgr5 polyclonal antibody (1:400, Abcam, UK), rabbit anti-AKT polyclonal antibody (1:500, Abcam, UK), and rabbit anti-VEGF polyclonal antibody (1:200, Abcam, UK). The primary antibodies were incubated overnight at 4 °C. Subsequently, the secondary antibody was incubated at room temperature for 1 h. After applying DAB color reagent (1:50, ZSGB-BIO, Beijing, China) for the color reaction, the results were observed under a microscope and the reaction was terminated promptly. Finally, the slices were sealed and statistical analysis was performed.

### 4.10. Total RNA Extraction

Total RNA was extracted using Trizol reagent (TAKARA, Kusatsu, Japan). The extracted RNA was reverse transcribed into cDNA using the TAKARA PrimerScriptTM RT Reagent Kit with gDNA Eraser (Perfect Real Time, Kusatsu, Japan). Primers targeting the gene of interest were designed and synthesized by Shanghai Biological Engineering Co., Ltd., Shanghai, China (Table 3).

### 4.11. Real-Time PCR Detection

The 10 μL reaction system included 5 μL of TB Green (TAKARA, RR820, Kusatsu, Japan), 0.5 μL of upstream primers, 0.5 μL of the downstream primers, 1 μL of cDNA, and an appropriate amount of double-distilled water to achieve a total volume of 10 μL. The reaction conditions were set as follows: 5 s at 95 °C, 30 s at 62 °C, and 30 s at 71 °C for a total of 35 cycles. Each experiment was repeated three times, and the final CT value of the target gene was obtained by calculating the average value.

### 4.12. IF Detection

The primary antibody used was mouse anti-Gli1 monoclonal antibody (1:500, Proteintech, Wuhan, China). Primary antibodies were incubated overnight at 4 °C. The secondary antibody (goat anti-mouse IgG, 1:10, Proteintech, Wuhan, China) was incubated at 37 °C for 1 h. After that, the sections were stained with DAPI for 10 min, sealed, and observed and photographed under a microscope. The entire experiment was conducted under dark conditions.

### 4.13. Single Cell RNA-Seq Data Processing

The FASTQ files of two samples (M + OSC group) were processed using the Cell Ranger (v.6.1.2) counting pipeline and the mouse reference genome version Mus_musculus to generate a feature barcode matrix corresponding to GRCm38.97.chr. The top 2000 highly variable genes (HVGs) were identified for integration. Data scaling and principal component analysis (PCA) were performed using default settings. Next, significant principal components (PCs) were selected using the elbow method, based on the standard deviation (SD) of PCs, for neighbor detection. Following this, Louvain clustering and Uniform Manifold Approximation and Projection (UMAP) analysis were conducted. The detection of subclusters also followed the same analysis pipeline.

For differential expression analysis in single-cell gene expression data, the DECENT software package determined statistical significance through likelihood ratio testing. All tests were two-sided, and a *p*-value < 0.05 was considered statistically significant.

### 4.14. Transcriptome Sequencing

FastQC (version 0.11.8) was used for the quality inspection of raw data. After passing quality control, the sequencing was carried out on a separate machine, where data quality control was completed during the process. A comprehensive quality assessment of the quantification and enrichment results for differentially expressed Gene Ontology (GO) terms was also performed.

DESeq2 (v.1.22.2) and edgeR (v.3.6.8) were used for differential expression significance analysis. The screening threshold was set to a false discovery rate (FDR) < 0.05 and a log2 fold change (log2FC) > 1 or <−1.

### 4.15. Quantitative Analysis of Protein Imprinting

Total protein extraction was carried out using the ProteinExt Mammalian Total Protein Extraction Kit from TransGen Biotech, Beijing, China. Protein quantification was performed with a BCA kit from the same manufacturer. The total protein was heated at 95 °C for five minutes to denature the proteins. In addition, 12% SDS-PAGE was utilized for the separation of proteins tagged with rabbit polyclonal antibodies against ATK (1:2000 dilution, Abcam, UK) and VEGF (1:1000 dilution, Abcam, UK). The resolved protein bands were transferred onto polyvinylidene difluoride (PVDF) membranes, which were subsequently blocked with a 5% skim milk solution at room temperature for one hour. The PVDF membranes were then incubated overnight at 4 °C with primary antibodies. After three TBST washes, the membranes were exposed to HRP-conjugated goat anti-rabbit IgG secondary antibodies at 37 °C for one hour. Detection of the target proteins was achieved using the ultra-sensitive ECL chemiluminescence kit from Proteintech, Wuhan, China.

### 4.16. Co-Culture Tissue Renal Subcapsular Transplantation

Nine 6–8-week-old female SCID beige mice were randomly divided into three groups (*n* = 3 each): the control group received no surgery, while co-cultured tissue masses were transplanted in the O + OSC and M + OSC groups. Mice were anesthetized by intraperitoneal injection of 1.25% Avertin (0.2 mL/10 g) and disinfected, and bilateral ovaries were removed through incisions at the costovertebral angles. After suturing and disinfection, mice were kept warm until recovery. Transplantation was performed 21 days later, at which point a dorsal incision was made, the renal fibrous membrane was opened, and the prepared co-cultured tissues were implanted. The incision was then sutured and disinfected, and mice were returned to the clean room for recovery with warmth maintenance.

### 4.17. Morphological Observation of Renal Tissue and Grafts After Transplantation

On the 21st day after allogeneic transplantation, the recipient mice were euthanized through carbon dioxide asphyxiation. The skin over the rib angle, superficial fascia, deep fascia, and muscular layer of the mice were quickly incised again, and the entire kidney containing the co-cultured tissue was extracted. The extracted kidney tissue and its surrounding capsule were immediately immersed in 4% PFA and fixed for 24 h. Samples from the control group and the two co-culture groups were collected and fixed simultaneously. Subsequently, paraffin sections were prepared and stained with HE for further microscopic observation and analysis.

### 4.18. Hormone Assay After Transplantation

On the 21st day after allogeneic transplantation, E2 levels were measured using ELISA. Three mice per group were used, and approximately 300 μL of whole blood was collected from the tail vein of each mouse, yielding approximately 150 μL of serum. Blood was stood at room temperature for 2 h, then centrifuged at 3000 rpm for 15 min at 4 °C to obtain the supernatant. The E2 ELISA kit (Wuhan Saiweier Biotechnology Co., Ltd., Wuhan, China; catalogue number: SEKM-0062; sensitivity: 1.0 pg/mL; detection range: 3.13–200 pg/mL; intra- and inter-assay CVs < 10% and <12%, respectively) was validated by the manufacturer for mouse serum. Standard, test, and blank wells were set up, and samples were added sequentially for washing, incubation with the test solution and substrate, and final machine detection.

### 4.19. Statistical Analysis

Data analysis was performed using SPSS 21.0 software, with the results presented as mean ± SD. Statistical analyses were conducted using *t*-test and one-way analysis of variance (ANOVA), and a *p*-value < 0.05 was considered statistically significant.

## 5. Conclusions

A co-culture of mouse mesonephros cells and OSCs in vitro led to the formation of follicle-like structures with basic follicular structural features and limited hormone secretion capacity. The mesonephros upregulates granulosa and theca cell-specific gene expression—functions that are closely related to the PI3K/AKT signaling pathway.

## Figures and Tables

**Table 1:** The number of follicles expressing green fluorescent protein at different culture times in M + OSC group (mean ± standard deviation) (*n* = 20).

Coculture Time	M + OSC Group
5th day	0.35 ± 0.49 ^bc^
14th day	16.0 ± 1.3 ^ac^
21st day	9.0 ± 3.45 ^ab^

Note: ^a^ = comparison with 5th day, *p* < 0.05; ^b^ = comparison with 14th day, *p* < 005; ^c^ = comparison with 21st day, *p* < 0.05. GFP-positive follicles indicate follicle-like structures containing mesonephros-derived GFP^+^ cells. Statistical significance was determined by one-way ANOVA followed by LSD post hoc test. Data are presented as mean ± SD (*n* = 20).

**Table 2:** The number of follicles in different time periods of co-culture (mean ± standard deviation) (*n* = 20).

Group	5th Day	14th Day	21st Day
OSC group	17.9 ± 1.68	27.9 ± 1.02	37.5 ± 1.47
M + OSC group	0.35 ± 0.49 ^a^	16.0 ± 1.3 ^a^	9.0 ± 3.45 ^a^

Note: ^a^ = comparison with OSC group, *p* < 0.05. Statistical significance was determined by two-way ANOVA followed by Tukey’s multiple comparisons test. Data are presented as mean ± SD (*n* = 20).

**Table 3:** Primer sequences used for real-time quantitative PCR.

Target Gene	GenBank Accession	Primer Sequence	Product Size (bp)	Annealing Temperature (°C)
Foxl2	NM-012020	F: ACAACACCGGAGAAACCAGAC R: CGTAGAACGGGAACTTGGCTA	145	62
Lgr5	NM-010195	F: CCTACTCGAAGACTTACCCAGT R: GCATTGGGGTGAATGATAGCA	165	60
Gli1	NM-010296	F: CCAAGCCAACTTTATGTCAGGG R: AGCCCGCTTCTTTGTTAATTTGA	130	61.2
GAPDH	NM-008084	F: AGGTCGGTGTGAACGGATTTG R: GACCCTGGCGTTCATTATGTC	123	62
AKT	NM-001165894	F:ATGAACGACGTAGCCATTGTG R:TTGTAGCCAATAAAGGTGCCAT	116	60
VEGF	MMU50279	F:CTGCCGTCCGATTGAGACC R:CCCCTCCTTGTACCACTGTC	233	61.2

## Data Availability

The authors confirm that all data generated or analysed during this study are included in this published article (and its Supplementary Information Files). The sequencing data provided by this study can be freely accessed in the scientific database (ScienceDB), and the website address is https://www.scidb.cn/en/s/AvuEf2 URL (accessed on 27 September 2026).

## Supplementary Materials

The following supporting information can be downloaded at: https://www.mdpi.com/article/10.3390/ijms27198719/s1.
