# Supplementary material for: Mouse Mesonephros Promotes Differentiation of Oogonial Stem Cells into Follicle-like Structures via the PI3K/AKT Signaling Pathway
Source: Int J Mol Sci. 2026 Sep 29;27(19):8719. doi: 10.3390/ijms27198719 (PMC13664789; doi:10.3390/ijms27198719)
Supplement: Supplementary file 1 [file ijms-27-08719-s001.zip › ijms-4499593-supplementary.pdf]

Supplementary Table S1. The number of follicles expressing green fluorescent protein at different culture times in M+OSC group (raw count)

Group cultivation number	point-in-time	The number of GFP-positive follicles
1	5th day	0
2	5th day	1
3	5th day	1
4	5th day	0
5	5th day	0
6	5th day	0
7	5th day	1
8	5th day	0
9	5th day	0
10	5th day	1
11	5th day	0
12	5th day	0
13	5th day	1
14	5th day	1
15	5th day	1
16	5th day	0
17	5th day	0
18	5th day	0
19	5th day	0
20	5th day	0
1	14th day	14
2	14th day	15
3	14th day	16
4	14th day	17
5	14th day	15
6	14th day	16
7	14th day	18
8	14th day	14
9	14th day	16
10	14th day	17
11	14th day	15
12	14th day	16
13	14th day	18
14	14th day	14
15	14th day	16
16	14th day	17
17	14th day	15
18	14th day	16
19	14th day	17
20	14th day	18

1	21st day	4
2	21st day	8
3	21st day	10
4	21st day	12
5	21st day	7
6	21st day	8
7	21st day	11
8	21st day	9
9	21st day	10
10	21st day	6
11	21st day	4
12	21st day	8
13	21st day	9
14	21st day	13
15	21st day	15
16	21st day	12
17	21st day	4
18	21st day	12
19	21st day	4
20	21st day	14

Supplementary Table S2. The number of follicles in different time periods of co-culture (raw count)

Group cultivation number	point-in-time	O+OSC group	M+OSC group
1	5th day	16	0
2	5th day	16	1
3	5th day	17	1
4	5th day	16	0
5	5th day	18	0
6	5th day	15	0
7	5th day	19	1
8	5th day	20	0
9	5th day	18	0
10	5th day	19	1
11	5th day	21	0
12	5th day	17	0
13	5th day	15	1
14	5th day	18	1
15	5th day	18	1
16	5th day	19	0
17	5th day	19	0
18	5th day	19	0
19	5th day	20	0

20	5th day	18	0
1	14th day	26	14
2	14th day	27	15
3	14th day	28	16
4	14th day	27	17
5	14th day	29	15
6	14th day	29	16
7	14th day	26	18
8	14th day	28	14
9	14th day	28	16
10	14th day	29	17
11	14th day	29	15
12	14th day	28	16
13	14th day	28	18
14	14th day	27	14
15	14th day	30	16
16	14th day	28	17
17	14th day	28	15
18	14th day	27	16
19	14th day	28	17
20	14th day	28	18
1	21st day	36	4
2	21st day	37	8
3	21st day	36	10
4	21st day	37	12
5	21st day	35	7
6	21st day	38	8
7	21st day	37	11
8	21st day	38	9
9	21st day	38	10
10	21st day	35	6
11	21st day	39	4
12	21st day	40	8
13	21st day	37	9
14	21st day	37	13
15	21st day	39	15
16	21st day	40	12
17	21st day	38	4
18	21st day	39	12
19	21st day	38	4
20	21st day	36	14
